# The Multifaceted Role of miR-21 in Pancreatic Cancers

**DOI:** 10.3390/cells13110948

**Published:** 2024-05-30

**Authors:** Clare Chen, Lusine Demirkhanyan, Christopher S. Gondi

**Affiliations:** 1 Department of Internal Medicine, University of Illinois College of Medicine Peoria, Peoria, IL 61605, USA; 2 Departments of Internal Medicine and Surgery, University of Illinois College of Medicine Peoria, Peoria, IL 61605, USA; 3 Departments of Internal Medicine, Surgery, and Health Science Education and Pathology, University of Illinois College of Medicine Peoria, Peoria, IL 61605, USA; 4 Health Care Engineering Systems Center, The Grainger College of Engineering, University of Illinois at Urbana-Champaign, Urbana, IL 61801, USA

**Keywords:** miR-21, pancreatic ductal adenocarcinoma, small non-coding RNA, proliferation, epithelial–mesenchymal transition (EMT)

## Abstract

With the lack of specific signs and symptoms, pancreatic ductal adenocarcinoma (PDAC) is often diagnosed at late metastatic stages, resulting in poor survival outcomes. Among various biomarkers, microRNA-21 (miR-21), a small non-coding RNA, is highly expressed in PDAC. By inhibiting regulatory proteins at the 3′ untranslated regions (UTR), miR-21 holds significant roles in PDAC cell proliferation, epithelial–mesenchymal transition, angiogenesis, as well as cancer invasion, metastasis, and resistance therapy. We conducted a systematic search across major databases for articles on miR-21 and pancreatic cancer mainly published within the last decade, focusing on their diagnostic, prognostic, therapeutic, and biological roles. This rigorous approach ensured a comprehensive review of miR-21’s multifaceted role in pancreatic cancers. In this review, we explore the current understandings and future directions regarding the regulation, diagnostic, prognostic, and therapeutic potential of targeting miR-21 in PDAC. This exhaustive review discusses the involvement of miR-21 in proliferation, epithelial–mesenchymal transition (EMT), apoptosis modulation, angiogenesis, and its role in therapy resistance. Also discussed in the review is the interplay between various molecular pathways that contribute to tumor progression, with specific reference to pancreatic ductal adenocarcinoma.

## 1. Introduction

Pancreatic ductal adenocarcinoma (PDAC) is a subtype of pancreatic cancer that is derived from exocrine tumors, accounting for 85% of all pancreatic cancers [1]. Common risk factors include diabetes, chronic pancreatitis, smoking, obesity, and hereditary syndromes [2,3]. Histologically, these malignant tumors have epithelial origins, forming gland-like structures [4]. Anatomically, the pancreas is difficult to visualize due to its retroperitoneal location [5]. Together with the lack of specific signs and symptoms [6], pancreatic cancer is often diagnosed at a late stage with local invasion or distant metastasis [6], making it ineligible for surgical resection [7]. Even for the minority of patients who may undergo curative surgery, the overall 5-year survival remains poor at approximately 12.5% [8,9]. In addition, cancer cells in PDAC can evade treatment and continue to grow, invade, and spread to the liver and lymphatics [10], which eventually proves fatal [11,12,13]. The current treatment options for PDAC are very limited. Surgery, the potentially curative therapy, is possible in only 15–20% of all patients [14,15]. Gemcitabine is a chemotherapy drug used that has been as the standard first-line treatment for advanced PDAC since the late 1990s. However, most patients demonstrate tumor progression with resistance to treatment after 3–5 months [12,13].

MicroRNA (miRNA) is a non-coding RNA that is short in length [16]. It suppresses gene expression by targeting the 3′ untranslated region (UTR) [17] on messenger RNAs (mRNAs) in various biological processes, such as cell differentiation, cell cycle, and cell death [18,19]. Emerging evidence has shown that aberrant expression of miRNAs can be a driving factor for certain types of cancer [20]. For example, some miRNAs are widely overexpressed in cancer cells and act as oncogenes, while others are usually lost in cancer cells and function as tumor suppressors [1,21,22]. Recent discoveries have identified miRNA as a potential cancer diagnostic tool and therapeutic agent [23]. By altering miRNA expression, studies have successfully attenuated growth in the adult brain tumor glioblastoma [24,25,26], instilling optimism in investigating miRNA in other solid tumors. With their role as a cancer regulator, miRNAs have been studied as potential cancer therapeutic agents [27,28]. Since miRNAs are more tissue-specific than other classically studied biomarkers, miRNAs are good candidates for targeted therapy [29,30]. However, miRNAs can also affect multiple genes at once with non-specific effects [31,32,33]. Thus, sophisticated gene sequencing is needed to unveil the complex genetic and epigenetic interaction between miRNAs and cancer progression. Studies in miRNAs are still considered a young field, and the effectiveness of using miRNAs as therapies to treat cancer needs further investigation [34]. With the swift development and decreasing cost of DNA sequencing technology, it is believed that genetic analysis of miRNAs can be utilized to generate patient-specific cancer therapy [35,36]. In face of these challenges, multiple microRNAs have been examined to identify their roles in PDAC [37].

### 1.1. Materials and Methods

To comprehensively examine the multifaceted role of miR-21 in pancreatic cancers, we employed a rigorous and systematic search strategy. We utilized prominent scientific databases like PubMed, Scopus, Web of Science, and Google Scholar. Our search terms encompassed “miR-21”, “microRNA-21”, “pancreatic cancer”, “PDAC” (pancreatic ductal adenocarcinoma), “diagnostic potential”, “prognostic potential”, “therapeutic implications”, and “biological functions”. We prioritized the recent literature, focusing on publications within the last decade while incorporating some seminal studies published earlier. Inclusion criteria narrowed the search to articles specifically investigating the role of miR-21 in pancreatic cancer. This included both original research and review articles published primarily in English. Studies that deviated from this focus or were duplicates were excluded. An initial screening of titles and abstracts was conducted to assess relevance, followed by a meticulous full-text review to ensure study quality and pertinence. The robustness of the experimental design, the sample size, and the significance of the findings were key factors in our quality assessment. From each selected article, we meticulously extracted critical information such as study objectives, methodologies, key results, and conclusions. Data pertaining to the diagnostic, prognostic, and therapeutic significance of miR-21 in pancreatic cancer, along with its underlying molecular mechanisms, were then synthesized to create a cohesive narrative. This meticulous approach ensured a thorough review, enabling us to present a well-informed and balanced perspective on the multifaceted role of miR-21 in pancreatic cancer.

### 1.2. Background of Pancreatic Ductal Adenocarcinoma

PDAC is found to arise from a series of precancerous pancreatic intraepithelial neoplasias (PanINs) [38]. The progression of normal pancreatic ductal epithelial cells into PanINs and adenocarcinoma is associated with alternation in expressions of oncogene and tumor suppressor genes [39], such as Kirsten Rat Sarcoma Viral Oncogene Homolog (KRAS), cyclin-dependent kinase inhibitor 2A [40,41] (CDKN2A), tumor protein p53 (TP53), and mothers against decapentaplegic homolog 4 (SMAD4). The activation of KRAS and the loss of function of TP53 and SMAD4 play important roles in stages of morphological changes and dysplastic evolution in PanINs [42]. Key histological features in the transformation of PanIN to adenocarcinoma include distortion in elongated epithelial tubules, invasion of the connective tissue matrix by atypical epithelial cells, and enlarged lumens filled with debris and inflammatory cells [43].

Modifications in the pancreatic extracellular matrix (ECM) and vasculature are commonly observed in PDAC. Through stimulation of stromal fibroblasts, there is an increase in PDAC ECM collagen and fibronectin content [44,45]. Studies have shown that ECM content correlates with overall survival among patients with PDAC [46], induces changes in tumor volume [47], and serves as a therapeutic target for PDAC [47,48]. In addition to ECM remodeling, angiogenesis is also observed in human PDAC tumors with elevated vascular endothelial growth factor (VEGF) in the pancreatic cancer epithelium [49]. Especially under hypoxic conditions, in vitro PDAC cancer cells increase VEGF expression to facilitate neoangiogenesis and promote tumor metastasis [50,51].

Though PDAC is associated with numerous molecular and cellular changes, we have yet to identify a biomarker that yields significance in detecting PDAC. In this review, we will discuss in depth the potential of miRNA as a diagnostic and prognostic marker for PDAC.

### 1.3. Importance of mir-21 in Cancer Research

Among various miRNAs, miR-21 has been overexpressed in many human cancers, contributing to the subversion of critical tumor suppressor pathways [52,53,54]. Through dysregulation of regular gene expression by overactive or underactive miRNAs, miR21 leads to tumor cell proliferation and cancer cell development [52,55,56]. For example, miR-21 can stabilize mature miR-499 post-transcriptionally, help suppress programmed cell death 4 (PDCD4), and subsequently promote cancer metastasis of head and neck squamous cell carcinoma [57]. In liver cells, the loss of miR-21 suppression by miR-122 leads to hepatocellular carcinoma [58]. Recently, studies have highlighted the vital role of miR-21 in the progression of pancreatic ductal adenocarcinoma (PDAC) [59,60,61]. By manipulating miR-21 expression, the offensive and defensive mechanisms against cellular stress and cell death signaling were found to have a profound effect on PDAC progression [15].

## 2. Mechanisms of mir-21 in Pancreatic Ductal Adenocarcinoma

By modulating cellular proliferation, apoptosis, invasion, and metastasis, miR-21 profoundly impacts cancer development. Several mechanisms have been studied to investigate the role of miR-21 in PDAC development. miR-21 induces cell proliferation by (1) inhibition of tumor suppressor genes [52] and upregulation of growth factors [56], (2) stimulation of endothelial-mesenchymal transition [62,63], (3) attenuation of apoptosis signaling pathways [64], and (4) disruption of angiogenesis [65].

In this section, we will discuss further the role of miR-21 on the growth of PDAC cells, specifically on cellular proliferation, EMT transition, apoptosis suppression, and angiogenesis (Table 1).

### 2.1. Regulation of Cell Proliferation

**Figure 1:**
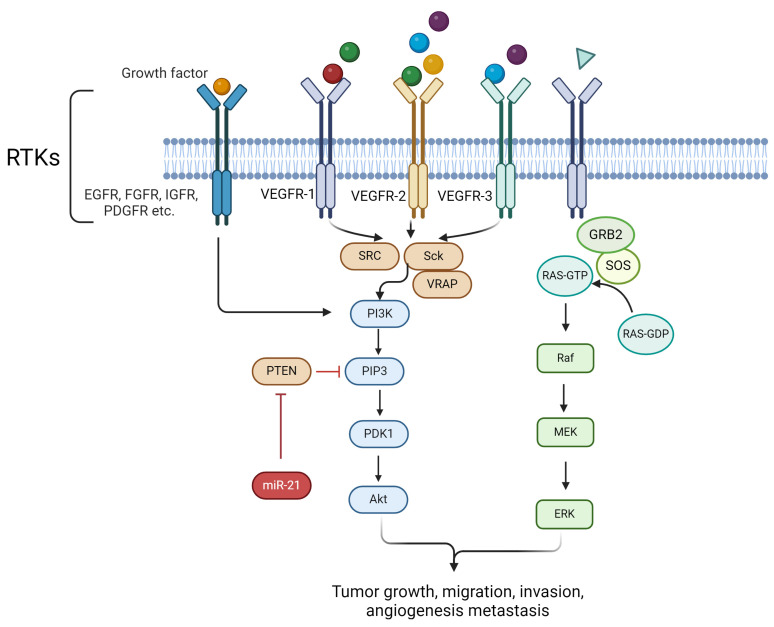
The Effect of miR-21 on the PTEN/AKT Signaling Pathway. By inhibiting PTEN, miR-21 indirectly promotes the AKT pathway to increase cell growth, angiogenesis, and cellular migration. (Figure created using Biorender.com accessed on 22 May 2024).

**Figure 2:**
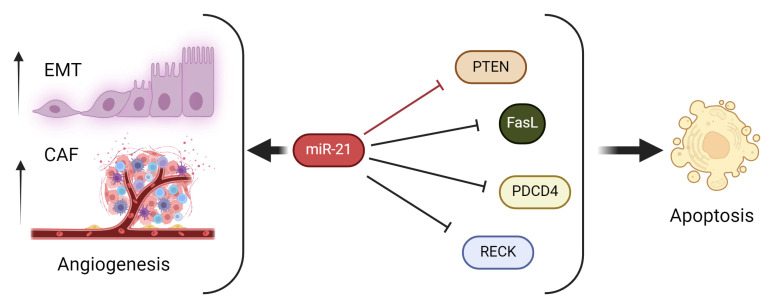
Role of miR-21 in Regulating Cell Proliferation. The increased expression of miR-21 promotes epithelial–mesenchymal transition, angiogenesis, and downregulation of apoptosis. (Figure created using Biorender.com accessed 22 May 2024).

miR-21 expression in PDAC appears largely regulated at the transcriptional level [66]. As a result, miR-21 expression is linked to cell cycle regulation. For example, it has been shown that phosphatase and tensin homolog (PTEN), a tumor suppressive protein, is one of the critical targets for miR-21 [67]. PTEN encodes a phosphatase that inactivates phosphatidylinositol 3-kinase (PI3K), antagonizing the action of PI3K, downregulating the Akt pathway, and inhibiting cell survival signals [68,69]. By suppressing PTEN, miR-21 indirectly activates the PI3K and AKT pathways and promotes cell proliferation. Numerous studies have demonstrated the inhibitory effect of miR-21 knockdown on PDAC cell growth, confirming the pro-proliferative role of miR-21 in cancer proliferation [70,71,72]. Besides PTEN inhibition, miR-21 also promotes cell proliferation by upregulating growth factors. For example, miR-21 increases the expression of vascular endothelial growth factor (VEGF) in the tumor microenvironment [73]. This allows the tumor to induce neovascularization, which is a crucial step for providing nutrients to support the primary tumor’s growth and facilitate metastasis [74] (Figure 1 and Figure 2).

### 2.2. Induction of Epithelial–Mesenchymal Transition

Epithelial–mesenchymal transition (EMT) is widely mentioned in the cancer literature [65]. EMT is a highly complex and dynamic process in which the cells lose their epithelial characteristics and gain mesenchymal properties [75]. This increases cellular motility and invasiveness, promoting metastatic behavior in cancer cells [76]. In the study by Xiao et al., the overexpression of miR-21 in cancer cells significantly upregulates EMT, stimulating cell migration and invasion [77]. Furthermore, miR-21 overexpression also downregulates Programmed Cell Death 4 (PDCD4) [78], supporting the role of miR-21 in the initiation and progression of EMT. It is important to note that EMT is an intricate process controlled by various pathways and molecular players, with miR-21 being one of many factors involved [79]. Recent studies have shown that other miRNAs, such as the miR-200 family, also play critical roles in the regulation of EMT. The miR-200 family suppresses EMT by promoting the expression of E-cadherin, an epithelial marker [80], and inhibiting the expression of mesenchymal markers [81]. This suggests the necessity of exploring the intricate interplay between different miRNAs and their collective impact on the EMT process in PDAC. By utilizing advanced technologies like bioinformatics and comprehensive in vivo analysis, the complex molecular landscape surrounding miR-21 and EMT will be unraveled, contributing to the advancement of therapeutic avenues for treating pancreatic cancer.

### 2.3. Modulation of Apoptosis

There are two major apoptotic pathways in mammalian cells: the intrinsic mitochondrial and extrinsic death receptor pathways. Both pathways converge on activating a family of proteolytic enzymes known as caspases [82]. The B cell lymphoma 2 (BCL-2) family of proteins mainly regulates the intrinsic mitochondrial pathway. This family comprises anti-apoptotic proteins, such as BCL-2 and BCL-XL, and pro-apoptotic proteins, such as BCL-10 and Bad [83]. The relative activities of the anti- and pro-apoptotic members of the BCL-2 family dictate whether apoptosis occurs or not [84]. The dysregulation of apoptosis signaling pathways profoundly affects cancer development [85]. Several genes involved in the apoptosis pathway have been identified as targets of miR-21 [64]. These genes include PTEN [33], PDCD4 [86], Fas ligand [87], reversion-inducing cysteine-rich protein with Kazal motifs (RECK) [88], and the tropomyosin gene [89]. It was shown that overexpression of miR-21 downregulates the expression of these target genes and inhibits apoptosis. In contrast, the knockdown of miR-21 in human cell lines has been shown to induce apoptosis [90]. The regulatory influence of miR-21 on apoptosis extends beyond the well-characterized caspase-dependent pathway. An additional layer of complexity is introduced by the caspase-independent cell death pathway mediated by the Apoptotic Inducing Factor (AIF) [91,92,93,94]. This protein resides within the mitochondrial intermembrane space. During apoptosis, AIF translocates to the nucleus, inducing DNA fragmentation and chromatin condensation, hallmarks of cell death, independent of caspases. This pathway can function in concert with the caspase-dependent pathway or independently under specific cellular stresses [91,92,95,96,97].

The current understanding of how miR-21 interacts with the AIF-mediated pathway remains to be fully elucidated. Future investigations into whether miR-21 influences AIF expression, translocation, or interacts with other regulators of this pathway are crucial for a comprehensive understanding of miR-21’s role in modulating apoptosis and its potential contribution to disease development.

### 2.4. Modulation of Autophagy

Autophagy is a critical cellular process involved in the degradation and recycling of cellular components, playing a pivotal role in maintaining cellular homeostasis. In pancreatic cancer, the modulation of autophagy is intricately linked to tumor progression, survival, and resistance to therapy [98,99,100,101,102]. However, the role of miR-21 in the pancreatic cancer autophagy pathway has yet to be reported. Interestingly, the downregulation of miR-216a-5p, miR-23b, miR-216a, miR-138-5p, miR-29c, miR-137, miR-421, miR-454, miR-372, miR-7, miR-29a, miR-9, and MIR506 was associated with increased autophagy and tumor progression in pancreatic cancer cells and patient samples [103]. Upregulation of these miRNAs inhibited autophagy through targeting autophagy-related genes, like ATG12, BECN1, ATG5, and ULK1, and increased sensitivity to chemotherapy drugs and radiation. On the other hand, upregulation of TSPAN1 promoted autophagy and was associated with a poor prognosis. The studies identified several autophagy-related miRNAs as potential prognostic biomarkers, therapeutic targets, and even delivery vehicles to increase chemosensitivity in pancreatic cancer by modulating autophagy; however, the direct role of miR-21 as a modulator of autophagy in pancreatic cancer remains unknown. A very recent article has discussed the role of Vacuole Membrane Protein 1 (VMP1) [104,105,106,107] an essential autophagic protein, and its relation to miR-21-mediated regulation [107]. Research has shown that miR-21 originates from a primary transcript located near the 3′-untranslated region of the VMP1 gene, further, the VMP1-miR-21 fusion transcript gets processed by Drosha, releasing mature miR-21 and the VMP1 mRNA transcript [106]. This could indicate that regulatory factors that modulate miR-21 could also modulate autophagy via VMP1, providing an indirect link between miR-21 and autophagy in pancreatic cancer.

### 2.5. Promotion of Angiogenesis

Angiogenesis is a strictly regulated process of forming new blood vessels from existing vasculature in response to damage and cellular repair. Dysregulation of angiogenesis contributes to the growth and invasion of pancreatic cancer by supplying nutrients for cellular proliferation [108]. Recent studies of miRNA on angiogenesis have revealed that increased expression of miR-21 promotes the proliferation of endothelial progenitor cells, leading to increased angiogenesis [109]. It has been shown that the inhibition of miR-21 results in a significant decrease in tumor size [110], attenuation of new tumor formation, and suppression of angiogenesis [111,112,113]. This suggests that the reduction in the density of micro-vessels formed within the tumor leads to nutrient depletion and cancer cell death. Research has also shown that miR-21 expression promotes angiogenesis by regulating VRAP [114] and the RAF-MEK-ERK [115] pathway. The increased expression of VEGF also positively correlates with miR-21 expression in PDAC [72,116]. Furthermore, in the tumor microenvironment study of pancreatic cancer, miR-21 was upregulated in activated fibroblasts and induced myofibroblast differentiation [65], promoting new vessel growth. In addition, miR-21 overexpression contributes to the upregulation of cancer-associated fibroblasts (CAFs), promoting PDAC desmoplasia and increasing resistance to gemcitabine therapy [117].

## 3. Clinical Implications and Importance of miR-21 in Pancreatic Ductal Adenocarcinoma

Given the crucial role of miR-21 in pancreatic cancer, many researchers are keen to explore the diagnostic value of miR-21 in PDAC [118]. A meta-analysis has shown that increased miR-21 levels are significantly associated with poor disease prognosis and reduced overall survival in PDAC patients, confirming the potential of miR-21 not just as a diagnostic marker but also as a prognostic indicator [119]. In addition to making the diagnosis, the level of miR-21 can reflect treatment efficacy in pancreatic cancer [120]. Patients with lower miR-21 expression after treatment have been found to have a longer median survival period compared to those with persistently high expression [121]. The diagnostic potential, prognostic value, and implications for therapeutic strategies indicate that the foundation of miR-21 in PDAC will continue to be a pivotal area of research, shaping both diagnostic and therapeutic paradigms in managing pancreatic ductal adenocarcinoma

### 3.1. Diagnostic and Prognostic Value of miR-21

Using miR-21 as a diagnostic biomarker for pancreatic cancer has been explored in several studies [122]. Through blood or saliva, miRNA levels can be collected with minimal invasion compared to traditional tissue biopsy [60,123]. Furthermore, the presence of exosomes prevents miRNAs from RNAse degradation, making miRNAs suitable candidates for biomarkers [124].

In comparing the plasma miRNAs between PDAC patients undergoing surgical resection and matched controls, miR-21 demonstrates a significant elevation in the PDAC sample group, which has the highest analytical performance with an area under the curve (AUC) > 0.99 [125]. Compared to chronic pancreatitis, the expression of miR-21 was also significantly elevated in PDAC samples [126]. Furthermore, the meta-analysis of miRNA profiling in pancreatic cancer showed four miRNAs, miR-21, miR-31, miR-210, and miR-155, are upregulated in pancreatic cancer. Among the four miRNAs, miR-21 is one of the most highly expressed in the serum of pancreatic cancer patients, with a sensitivity of 0.7 and a specificity of 0.80 [127]. This evidence suggests the high diagnostic value of circulating miR-21 in pancreatic cancer.

Though these studies demonstrated the diagnostic potential of miR-21 in pancreatic cancer using blood-based miRNA profiling, data from larger population sizes and different stages of pancreatic cancer are required for further validation. Furthermore, the diagnostic value of saliva miR-21 analysis is still under investigation [128,129]. If the diagnostic potential of miR-21 continues to be validated in subsequent studies, it could be used in clinical settings as a non-invasive and efficient method for detecting PDAC.

Several retrospective studies have investigated the importance of miR-21 as a prognostic biomarker. The results have shown that high levels of miR-21 expression are associated with significantly decreased overall survival and disease-free survival in PDAC patients [121,130]. For instance, in the study of 76 patients diagnosed with pancreatic cancer, Ali S. et al. identified that increased miR-21 was significantly associated with worse survival and chemotherapy resistance [131]. In another study by Abue et al., increased plasma miR-21 expression in PDAC patients was also associated with advanced-stage lymph node metastasis, liver metastasis, and shorter overall survival [132]. Furthermore, in an international multicenter study of 686 PDAC patients, miR-21 was analyzed using tissue microarrays and correlated with clinical pathologies. The miR-21 overexpression was significantly associated with increased tumor size (>2 cm) and lymph node metastasis [121].

Thus, it can be suggested that miR-21 can be used as an adjunct prognostic factor and may help identify patients needing more aggressive adjuvant therapy. The findings from these trials can significantly impact the current treatment methodology, which will be discussed in the next section.

### 3.2. Therapeutic Targeting of miR-21

Gemcitabine is a standard chemotherapy used in treating PDAC and is a prodrug that prevents DNA synthesis by inhibiting the enzyme ribonucleotide reductase [133,134]. While many patients with PDAC develop resistance to gemcitabine [12], a study has shown that the combination of anti-miR-21 and gemcitabine produces a more effective treatment response than gemcitabine alone [135]. A novel approach to anti-miR-21 is using antisense oligonucleotides complementary to miR-21, thereby preventing the miRNA from exerting its oncogenic effects [136]. In a preclinical study using a xenograft model of PDAC, treatment with anti-miR-21 oligonucleotides was shown to reduce tumor growth and increase survival [137]. These discoveries suggest that anti-miR-21 therapy might also help to overcome chemoresistance.

Two major small RNA-based anti-miR-21 therapy types have been developed in recent years, including ribozyme transgenes and locked nucleic acid (LNA) oligonucleotides. The former enables target-specific degradation of miRNA in a catalytic fashion. At the same time, the latter addresses the intrinsic shortcomings of DNA oligonucleotides, such as their poor binding affinity and nuclease sensitivity [138,139]. In PDAC, miR-21 knockdown by anti-miR therapy has been shown to effectively suppress tumor growth and increase the sensitivity of cancer cells to chemotherapeutic drugs [140]. Specifically, the downregulation of miR-21 expression in PDAC cells has increased apoptosis and reduced cell viability [141]. It also enhances tumor sensitivity to the cytotoxic effect of gemcitabine, leading to a more significant suppression of cell growth [142]. These research findings indicate that targeting miR-21 could be an effective treatment strategy for PDAC. Although further research is required to optimize the therapeutic benefits of anti-miR-21 treatment and to develop effective delivery methods, the initial success made in preclinical studies is promising. It has shed light on novel PDAC therapy developments.

Another way to target miR-21 is to develop small-molecule inhibitors against the biogenesis of miR-21, blocking the production of pre-miR-21 or its maturation pathway [143]. While this approach is challenging, a recent study found that antagomir was able to block miR-21 in the cytoplasm and nucleus while contributing to the degradation of miR-21 and inhibiting angiogenesis, which is a hallmark of cancer progression [144]. Recent studies highlight that miR-21 regulates many important biological processes [145]. Thus, it is critical to investigate the precise molecular mechanisms of action between miR-21 and its downstream targets in different cell types or disease states to open new avenues for developing miR-21-based therapies for PDAC.

## 4. miR-21 and Resistance to Therapy in Pancreatic Ductal Adenocarcinoma

Chemoresistance in PDAC is a significant obstacle to successful treatment. It has been shown that miR-21 is significantly upregulated in gemcitabine-resistant cell lines [131]. In addition, the knockdown of miR-21 can re-sensitize these cells to gemcitabine by inducing apoptosis and inhibiting cell growth [146]. Among patients undergoing surgical resection for PDAC, high miR-21 expression was significantly associated with shorter disease-free survival and overall survival in patients receiving adjuvant gemcitabine [147]. Similarly, radio-resistance in PDAC is also a critical problem. miR-21 is shown to be involved in the regulation of DNA damage response and repair in PDAC. A study has found that inhibition of miR-21 can sensitize PDAC cells to radiation by increasing DNA damage, leading to cell cycle arrest and apoptotic cell death [148].

Targeted therapy is a form of cancer treatment that focuses on genes and proteins involved in cancer cell proliferation and survival [149]. However, patients with PDAC often develop resistance to targeted therapy drugs [150,151,152]. Studies found that the PI3K-AKT pathway, the downstream target of miR-21, was significantly upregulated in erlotinib-resistant PDAC cells [153,154]. It has also been demonstrated that miR-21 affects the response to erlotinib by downregulating the expression of PTEN [155]. Similarly, miR-21 also affects cancer cells’ response to cetuximab [156] and can potentially overcome the KRAS mutation, a major genetic cause of cetuximab resistance [157]. These findings suggest that miR-21 plays an active role in resistance to standard PDAC treatments, including chemotherapy and targeted therapy. It is crucial to investigate whether combining miR-21 inhibitors with different treatment modalities can enhance therapeutic efficacy in PDAC. Moreover, considering the potential of miR-21 as a predictive biomarker for therapy response in PDAC, future clinical trials are warranted to test miR-21 inhibitors in subgroups of patients selected based on their miR-21 expression profile.

### 4.1. Role of miR-21 in Chemoresistance

Among the ongoing progressions in PDAC, miR-21 has developed as a key player in chemoresistance [131]. The intrinsic and acquired characteristics of chemoresistance allude to the decreased affectability of diseased PDAC cells to chemotherapy [158]. In intrinsic resistance, therapy failure results from an existing genetic alteration before initiating treatment. On the other hand, acquired resistance occurs after drug treatment has been administered [159]. Acquired chemoresistance is often observed in PDAC and treated with gemcitabine. Several studies have investigated the mechanism of drug-induced chemoresistance in PDAC. For example, alterations in the regulator of EMT and hyperactivity of EKR, which downregulate pro-apoptotic Bax protein [160], are observed in gemcitabine-resistant PDAC cell lines [161]. Furthermore, the gemcitabine-resistant PDAC also demonstrated resistance to vesicular stomatitis virus (VSV) therapy [162]. The expression of miR-21 overrides the action of GAS5, a long-non-coding RNA that suppresses pancreatic cell proliferation, in gemcitabine-resistance PDAC [135]. This evidence reveals the various pathways through which miR-21 influences the evolution of PDAC cells in response to chemotherapy.

The mechanisms of intrinsic chemoresistance are different and complex. However, the role of miR-21 as a characteristic driver of chemoresistance has been perceived. The potential role of miR-21 in chemoresistance was identified in prostate malignant growth [163,164]. Since then, the relationship between miR-21 and chemoresistance has been detailed in various solid tumors and hematological malignancies. Late examinations have uncovered that miR-21 DNA hypomethylation is associated with pancreatic cancer [165], showing that acquired epigenetic alterations by miR-21 could be endowed from both parenteral alleles. It is perceived that miR-21 can independently modulate and actuate numerous oncogenic pathways. For instance, a significant increase in miR-21 expression was observed in gemcitabine [166], oxaliplatin [167], and erlotinib-resistant cell lines [141] when compared to the drug-sensitive cell lines. The increase in miR-21 is further associated with decreased PTEN and PDCD4 expression in drug-resistant lines [131].

In addition to miR-21 and its downstream target, the recent literature also examines the role of miR-21 carriers, such as extracellular vesicles (EVs), in chemoresistance [168]. By delivering chemoresistance-inducing miRNA to recipient cells, EVs facilitate the development of chemotherapy resistance in cancer cells [169,170,171]. More specifically, the exosomal miR-21 was elevated in metastatic breast cancer [172] and was responsible for inducing cisplatin resistance in ovarian cancer cell lines [173]. In PDAC, the long-term exposure to gemcitabine increased miR-155 and miR-21 expression, which also increased the secretion of miR-155 and miR-21 exosomes from cancer-associated fibroblasts (CAFs) to other PDAC, contributing to the development of gemcitabine resistance [13,174]. Late investigations have indicated that the hypoxic tumor microenvironment also induces the secretion of exosomal miRNA, favoring PDAC proliferation and evasion from chemotherapy [175,176]. Recently, Angel and colleagues have presented evidence that hypoxia contributed to the overexpression of miR-21 in prostate tumors [177]. Considering the upregulation of miR-21 expression in hypoxic conditions, it is possible to propose that overexpression of miR-21 extracellular vesicles could mitigate chemoresistance in PDAC.

### 4.2. Impact of miR-21 on Radio-Resistance

Radiotherapy remains one of the most effective treatments for many cancer patients. By inducing DNA damage, radiation activates various molecular signaling mechanisms responsible for regulating cell cycle progression, such as repairing damaged DNA or inducing apoptosis [178]. However, the median survival of patients treated with radiotherapy varies significantly [179,180]. Such disparity may be explained by the difference in radiation dose and mechanisms of radio-resistance [181,182,183,184]. The effect of miR-21 on radio-resistance has been studied in several tumors. It was found that the radiosensitivity of cancer cells was affected by the level of miR-21 [185]. For example, in an in vitro cell culture study of colorectal cancer, the overexpression of miR-21 led to a lower apoptotic index than the control group. As a result, the cell survival fraction after radiation was much higher, indicating that the overexpression of miR-21 decreased radiation efficiency [186]. On the other end, a study on esophageal squamous cell cancer found that the suppression of miR-21 increased the sensitivity of cancer cells to radiation [187]. In another study of head and neck cancer cells, the group that received anti-miR-21 showed a significant reduction in tumor volume compared to the radiation-only group [188]. These findings indicate that the downregulation of miR-21 significantly reduces the tumor growth rate after treatment with radiation, suggesting that combining radiation and anti-miR-21 could improve the treatment outcome.

### 4.3. miR-21-Mediated Targeted Therapy Resistance

The discovery that miR-21 is involved in targeted therapy resistance has significantly catalyzed the development of miR-21 inhibitors as a potentially effective cancer treatment. In the United States, erlotinib, an epidermal growth factor receptor (EGFR) inhibitor, is the only targeted therapy approved for PDAC by the Food and Drug Administration. When combined with gemcitabine, erlotinib has been shown to improve the overall survival of patients with metastatic PDAC [189]. However, erlotinib resistance is also observed in pancreatic cancer [190]. While resistance to targeted therapy poses a further challenge in treating PDAC, other studies have shown the role of miR-21 inhibitors in reversing drug-resistance characteristics in cancer cells. In non-small cell lung cancer, the efficacy of the EGFR inhibitor in reducing tumor size was significantly enhanced when accompanied by a miR-21 inhibitor. This highlights the ability of anti-miR-21 to reverse targeted therapy resistance in cancer cell lines [191]. This finding is highly relevant for PDAC treatment development, as EGFR inhibitor-based therapy (e.g., erlotinib and lapatinib) is a significant direction of targeted therapy research for PDAC [192]. By using miR-21 inhibitors as modulators, it is hoped to develop novel therapies to suppress tumor resistance in PDAC.

The targeting of miR-21 represents a relatively new but rapidly growing treatment strategy for combating the resistance of PDAC. By exploiting miR-21’s significant role in regulating therapy resistance pathways and tumor suppressor genes [193] in drug-resistant cell populations, miR-21 inhibitors have shown promise in future PDAC-targeted therapy regimens. Further investigating the interplay between miR-21 and its targets can provide researchers with a comprehensive picture of miR-21’s potential usage in multimodal therapy, such as a combination of oncolytic viral therapy [194] and conventional chemo-radiation [195,196]. Through unraveling the complex miR-21 regulatory network and rationally designing effective combinatory strategies with miR-21 inhibitors, the future of PDAC-targeted therapy is expected to arrive at the era of precision medicine, in which therapy treatment can be customized and patient-specific.

## 5. Interplay between Life-Saving Biomarkers and miR-21 Expression in Pancreatic Ductal Adenocarcinoma

**Figure 3:**
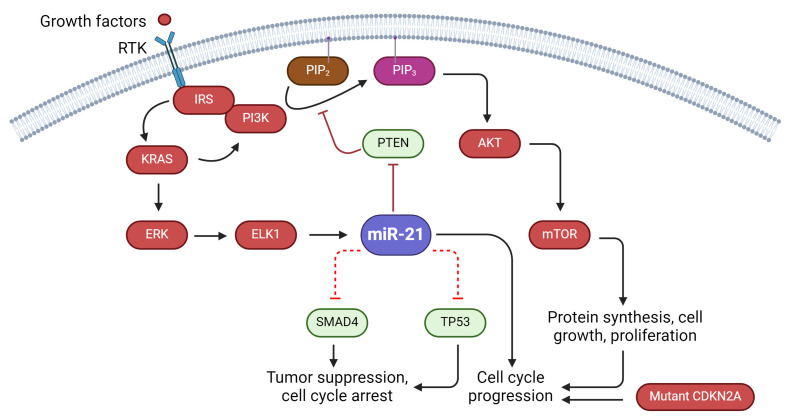
Relationship of miR-21 with KRAS, PTEN, SMAD4, and TP53. The expression of ELK1 enhances miR-21 expression, which leads to downregulation of SMAD4, PTEN, and TP53, inhibiting tumor suppressive functions. The increased expression of miR-21 and mutant CDKN2A promotes cell cycle progression and cellular progression. (Figure created using Biorender.com accessed 23 May 2024).

In this section, the relationship between miR-21 and known biomarkers, such as KRAS oncogene, tumor protein P53 (TP53), cyclin-dependent kinase inhibitor 2A (CDKN2A), and mothers against decapentaplegic homolog 4 (SMAD4), also known as the “Big Four”, will be discussed [197]. The primary argument revolves around the collaboration between miR-21 and the RAS pathway [198], along with point mutations in the KRAS oncogene, the loss of function mutations in tumor suppressor CDKN2A, and the inactivation of the tumor suppressor gene SMAD4 [197], all of which contribute to PDAC progression (Figure 3).

### 5.1. miR-21, KRAS Mutations: Impact and Significance

RAS proteins are GTPases that promote cellular proliferation and survival through a series of signaling cascades [199]. The gain-of-function Ras protein was one of the first oncogenes to be discovered in the 1980s and has been the subject of thousands of studies [200], especially in the development of PDAC [201]. The activation of the KRAS oncogene is well recognized as the earliest genetic event in the progression of PDAC [202]. In at least 95% of all PDAC cases, the KRAS gene is mutated [202,203]. More specifically, the point mutation in the KRAS oncogene, a member of the RAS protein family, is a critical contributor to the early oncogenic transformation in PDAC [204,205]. Such activation mutations stimulate the PI3K/AKT/mTOR pathways, favoring cell proliferation and survival [206].

The regulation between the KRAS mutation and miR-21 has also been established. In non-small cell lung cancer, the overexpression of KRAS and its downstream transcription factor, ELK1, upregulates miR-21. Furthermore, the positive regulatory feedback loop between miR-21 and members of the KRAS mutant in ovarian cancer illustrates the critical role of miR-21 in cancer cell proliferation [207]. With no known anti-cancer gene in the RAS pathway, the interaction between miR-21 and KRAS highlights its significance as a target for therapeutic intervention and cancer prognosis.

### 5.2. Oncogenic Role of miR-21 and TP53 Alterations in Cancer

Another important aspect of miR-21 is its relationship with the tumor protein P53 (TP53). TP53 is a tumor suppressor gene that induces cell cycle arrest, autophagy, and apoptosis [208,209]. With loss-of-function mutations, the TP53 mutant leads to uncontrolled tumor cell proliferation [210]. In pancreatic cancer research, TP53 is especially significant, as almost 70% of all pancreatic cancer cases involve mutations of the TP53 gene [211]. It is suggested that the tumor-promoting activities of miR-21 and the suppression of the TP53 gene synergize in the development of PDAC. Previous experiments have shown that TP53-induced apoptosis can be suppressed by miR-21 [212], adding another dimension to the anti-apoptotic property of miR-21.

Moreover, in the presence of miR-21, the migratory effect induced by TP53 inhibition is enhanced [213], strengthening the argument that miR-21 and TP53 alterations contribute to the aggressiveness of PDAC. In addition, miR-21 has been found to be overexpressed in cells with TP53 mutations, where even more tumor-promoting abilities are seen compared to the wild-type TP53 [214]. These findings suggest that TP53 alterations may lead to the upregulation of miR-21 and imply the possibility of using miR-21 as a biomarker to predict TP53 mutational status in the tumor. While the overexpression of miR-21 alone has already been confirmed to be related to poor prognosis in PDAC patients [118], the prognosis result is even worse in those with both miR-21 overexpression and TP53 mutations [214]. This may lay the foundation for adopting miR-21 expression and TP53 mutational status as a combined biomarker for cancer. All in all, the interactions between miR-21 and the TP53 gene provide another testament to the important role miR-21 plays in PDAC and create new possibilities for future therapies targeting miR-21.

### 5.3. Association between miR-21 and Tumor Suppressor Genes

In addition to KRAS and TP53 proteins, miR-21 also has a significant interaction with tumor suppressor genes. A loss of function mutation in p16/CDK inhibitor 2A (CDKN2A) has been identified in PDAC. The mutant CDKN2A overly activates cyclin-dependent kinases 4 and 6 (CDK4/6), which promote G1 cell cycle progression [211]. On the other hand, miR-21 is also responsible for promoting cell proliferation at the S phase of the cell cycle [215]. In a recent in vitro experiment, the combination of miR-21 and CDK4/6 inhibitors was shown to reduce cell proliferation and increase apoptosis in PDAC cell lines [216]. In the face of chemo and radiotherapy resistance in PDAC, this novel finding brings hope to novel therapeutic regimes for treating PDAC.

While the CDKN2A mutant contributes to cell-cycle dysregulation, loss of function in SMAD4 contributes to the metastatic characteristic of PDAC [217]. Though the relationship between SMAD4 and miR-21 in PDAC has not been established, recent studies have shown that miR-21 inhibits the SMAD4 signaling pathway in hepatitis [218] and rheumatoid arthritis [219]. By suppressing SMAD4, miR-21 consequently promotes fibrogenesis through activation of matrix metalloproteinases (MMP), a characteristic observed in chemo-resistant PDAC [117].

Aside from the “Big 4’s”, PTEN is another important tumor suppressor gene that interacts with miR-21 during the progression of PDAC [220]. By binding to the 3′UTR on PTEN, miR-21 suppresses PTEN translationally and downregulates PTEN tumor suppressor function [221]. This reflects a study that found increased miR-21 expression and decreased PTEN expression in patients’ PDAC tissues [11]. These findings were also supported by both in vitro and in vivo studies. For example, a study using immunohistochemistry staining has found a negative correlation between miR-21 and PTEN expression levels in tumor samples [222]. Conversely, PTEN-related pathways for apoptosis and cell proliferation were found to be downregulated by miR-21, as demonstrated in in vitro tissue culture studies [223]. All these data support the tumor suppressive role of PTEN in PDAC and reflect the significance of miR-21’s regulation of PTEN.

The specific interaction between miR-21 and PTEN serves as a nodal point in various molecular pathways of PDAC. For instance, PTEN can suppress the activation of the Protein Kinase B (PKB or AKT) and Extracellular Signal-Regulated Kinase (ERK) pathways, which are both essential to cell survival, proliferation, and invasion [224]. Indeed, studies have found that miR-21 contributes to an increased proliferation of PDAC cells and a stronger resistance to apoptosis induction, mainly due to the over-activation of the AKT pathway due to PTEN suppression [116]. This makes miR-21 an exciting target for potential novel molecular therapy. By inhibiting the function miR-21, we can artificially ‘upregulate’ PTEN, leading to an increased apoptosis rate and decreased invasiveness of PDAC cells. Though there might be potential limitations, such as the off-target effects of miR-21 inhibitors, such therapy is believed to show promising future results. It is important to carry out more in-depth research and explore the possibilities. By then, the author believes that one day, miR-21 inhibitors could be used with current therapies to enhance treatment response and perhaps reduce chemoresistance in advanced PDAC patients (Table 2).

## 6. Future Perspectives, Research Directions, and Potential Areas of Investigation

With the advancement of biotechnologies, the cancer research community can enhance our current understanding of miRNAs. Through RNA sequencing, researchers can perform genome-wide identification of small non-coding RNAs and study their expression patterns [225]. Instead of microarrays, RNA-sequencing can perform gene signature clustering [226] and tumor classification [227]. In the near future, we anticipate uncovering miR-21’s regulatory network and identifying different clusters and gene signatures of different PDAC molecule subtypes. Moreover, new bioinformatics techniques can be developed to identify key targets and essential pathways regulated by miR-21. Currently, only a limited number of tools are designed to predict targets regulated by specific miRNAs [228], and there is no absolute gene prediction tool for miR-21. Third, treatment delivery methods need to be optimized to facilitate the accurate delivery of miRNA inhibitors to target tissues. Currently, the most promising method is local delivery techniques, such as direct intratumoral injection. However, there is a lack of a safe and effective systemic delivery approach in clinical practice [229]. Finally, the development of miRNA inhibitors in clinical research should be accelerated. Though anti-miRNA oligonucleotides and locked nucleic acids are used in laboratory research [230,231,232], translating basic research into clinical practice is relatively slow. In the future, the application of miRNA inhibitors as a therapeutic approach in PDAC should be broadly investigated, and corresponding clinical trials should be carried out. Once miR-21-targeted therapy is established, it can be used as an adjuvant therapy in treating PDAC and potentially enhance current chemotherapeutic efficacy.

### 6.1. Unraveling the Complex Regulatory Network of miR-21

To fully understand the diverse effects of miR-21 on PDAC, it is critical to map out the complex regulatory network of miR-21. This could be achieved by studying the transcriptional and post-transcriptional regulators of miR-21. One of the most critical steps in miRNA biogenesis is the processing of the primary transcript (pri-miRNA) into a shorter precursor (pre-miRNA) by the Drosha/DGCR8 complex [233]. RNA activation proteins, such as TARBP2, could enhance the Dicer-elicited miRNA maturation process [234]. Bioinformatics analysis has predicted the presence of several putative binding sites for various transcription factors (TFs), including NF-kB, in the miR-21 promoter region [235]. In severe pathophysiological conditions with overwhelming TLR/IL-1 signaling, miR-21 expression was found to be positively regulated by NF-kB through the TLR/IL-1/mal/MyD88 pathway [54].

In addition to the upstream regulation of miR-21, the inhibitory role of miR-21 on tumor suppressor genes is another area of significance in cancer research. For example, miR-21 inhibits PTEN, PDCD4 [53], and RECK [236] by complementary binding to the 3′UTR on the target mRNA [237], inhibiting translation and downregulating tumor suppressor functions. With a luciferase reporter assay, researchers could monitor miR-21 activity by inserting the target 3′UTR construct onto the luciferase reporter vector. Despite having a short nucleotide sequence, miR-21 can exert its carcinogenic effect on various targets, illustrating the diverse and complex effect of miR-21 in pancreatic cancer development.

With tremendous progress in unveiling the regulatory network of miR-21 in animal models, it will be beneficial to translate these laboratory findings into therapeutic interventions and investigate the clinical significance of miR-21 in PDAC progression and treatment.

### 6.2. Exploring Potential Synergies by Combining miR-21 Inhibitors with Other Therapies

The recent discovery that miR-21 is present in exosomes opens a novel way of delivering targeted molecular therapy against PDAC [238]. Exosomes are cell-derived vesicles secreted by many cells, including cancer cells, and found in bodily fluids such as blood and urine [239]. It has been shown that exosomal miR-21 is bioactive and can modify gene expression in recipient cells [240]. Exosomes can also interact with the extracellular matrix and attract specific cell types [241,242]. This suggests that the miR-21 payload in exosomes could be delivered to specific cellular compartments within the pancreas to modify the tumor microenvironment by modifying the stromal cells. Emerging methods, such as gene editing technology and nanoparticle-based delivery systems, could potentially block the oncogenic activity of miR-21 in the tumor microenvironment [243]. Through the nanoparticle-based delivery system, anti-miRNA oligonucleotide therapy inhibited tumor growth in pancreatic, gastric, and liver cancer [244]. Furthermore, drug development for specific targeting of exosomal delivery pathways is intensively studied in many fields [245,246]. Together with improved methods of exosome delivery system, miR-21-based exosomal gene therapy can potentially open a new opportunity for therapeutic intervention in PDAC.

Moreover, the availability of more sophisticated mouse models for pancreatic cancer that accurately recapitulate the progression and molecular subtypes of PDAC in humans will be invaluable for in vivo validation studies of miR-21 inhibition [247]. Such models include genetically engineered mouse models [248] and patient-derived xenografts [249,250]. With the emergence of novel therapeutics, it is promising that the detailed study targeting miR-21 in cancer therapeutics will translate to the clinical arena, offering more options for patients suffering from this devastating malignancy.

### 6.3. Investigating the Role of miR-21 as a Biomarker for Treatment Response

While miR-21 has the potential to be a prognostic biomarker for PDAC [118], miR-21 expression is also associated with treatment response in PDAC. A retrospective study has identified that the reduced miR-21 expression among PDAC patients treated with adjuvant chemotherapy was associated with better survival outcomes [251]. Another study also demonstrated that patients with higher miR-21 expression had significantly shorter overall survival despite the completion of adjuvant therapy. In the same study, elevated miR-21 expression also correlates with characteristics of gemcitabine resistance in PDAC, suggesting miR-21’s role in chemoresistance [116]. Though the precise mechanism between miR-21 and PDAC therapy resistance remains unclear, these findings indicate the significance of miR-21 as a potential biomarker in PDAC disease and treatment monitoring. Thus, further investigation into the molecular interaction between miR-21 and PDAC is hoped to improve the accuracy and efficacy of predicting PDAC progression to enhance patient survival outcomes and quality of life.

## 7. Concluding Remarks

miR-21, commonly overexpressed in various tumor types, is found to hold significant importance in promoting cancer development and progression in multiple ways, including enhanced cell proliferation, migration, and invasion, as well as resistance to cell death and induction of angiogenesis [252]. In the context of PDAC, numerous lines of evidence collectively support the central role of miR-21 in mediating many aspects of PDAC development and progression, making it a promising diagnostic, prognostic, and therapeutic target [118]. In this regard, the complexity of miR-21’s roles in PDAC showcases the necessity and opportunities to explore the multifaceted regulatory activities of miR-21 in PDAC to strategize tailored therapy. Sophisticated interdisciplinary efforts integrating in-depth biological knowledge and cutting-edge biotechnology have elucidated our view of microRNAs in human cancer [253,254]. We have considerably advanced our understanding of miR-21 in PDAC over the past few years with newly developed genetic mouse models [247] and revolutionary genome editing techniques [255].

It is evident that miR-21 plays an essential, multifaceted role in pancreatic cancers. And our understanding of the impact of miR-21 in pancreatic cancers has gone far beyond a certain level of biological findings, especially when this review sheds light on miR-21’s impact on therapeutic resistance. By discussing the molecular mechanisms and reflections on different research approaches, the review has given a comprehensive picture of miR-21 in pancreatic cancer. As we have summarized in the future perspectives and research directions, miR-21 continues to be a promising subject for PDAC research. And with the increasing knowledge of this small RNA molecule, we are one step closer to developing effective miR-21-based therapies (Table 1).

Nonetheless, there are still gaps in our knowledge on several issues regarding PDAC, including the primary cell of origin, the stepwise accumulation of genetic alterations, and the evaluation of interventions in suppressing PDAC development and progression. Finally, the reprogramming effect of miR-21 on the microenvironment of PDAC and tumor metabolism demands our attention. This will not only improve our understanding of PDAC biology but also shed light on the development of novel, effective therapeutics.

**Table 1:** Summary of the gene targets of miR-21 associated with PDAC.

Effect of miR-21 on Target	Target Genes and Proteins	Function of Genes and Protein	Pathway Involved	Main Result	References
**Downregulate**	PTEN	Tumor suppressor	PI3K/AKT/mTOR	Cell proliferation, chemoresistance,	[66,68,69,153,154]
**Upregulate**	VEGF	Angiogenesis	AKT/ERK SRC/PI3K/AKT/mTOR	Angiogenesis	[73,112,116]
**Downregulate**	PDCD4	Tumor suppressor	Fibroblast differentiation	Epithelial–mesenchymal transition, angiogenesis	[65,78,117]
**Downregulate**	PDCD4, Fas ligand, RECK, and tropomyosin	Apoptosis	Caspase-dependent apoptosis	Cell proliferation	[78,86,87,88,89]
**Upregulate**	ERK	Apoptosis	ERK1/2-Bax/Bcl-2	Gemcitabine resistance	[160,161]
**Downregulate**	SMAD4	Tumor suppressor	Transcription factor	fibrogenesis	[256,257]

**Table 2:** The effect of miR-21 on oncogene and tumor suppressor gene mutations in PDAC.

Effect of miR-21 on Target	Target Genes	Function of Genes and Protein	Pathway Involved	Main Result	References
**Gain of function**	KRAS	Oncogene	PI3K/AKT/mTOR	Cell proliferation	[206]
**Loss of function**	TP53	Tumor suppressor	Cell cycle arrest, autophagy, and apoptosis	Cell proliferation	[208,212]
**Loss of function**	CDKN2A	Tumor suppressor	Cell cycle regulation	G1 cell cycle progression	[211]
**Loss of function**	PTEN	Tumor suppressor	PI3K/AKT/mTOR	Cell proliferation	[66,68,69,153,154]

## Data Availability

This is a review article, and no new data were generated here.
